# Novel Methods for Measuring Depth of Anesthesia by Quantifying Dominant Information Flow in Multichannel EEGs

**DOI:** 10.1155/2017/3521261

**Published:** 2017-03-16

**Authors:** Kab-Mun Cha, Byung-Moon Choi, Gyu-Jeong Noh, Hyun-Chool Shin

**Affiliations:** ^1^Department of Electronic Engineering, Soongsil University, Seoul, Republic of Korea; ^2^Department of Clinical Pharmacology and Therapeutics, Asan Medical Center, Seoul, Republic of Korea

## Abstract

In this paper, we propose novel methods for measuring depth of anesthesia (DOA) by quantifying dominant information flow in multichannel EEGs. Conventional methods mainly use few EEG channels independently and most of multichannel EEG based studies are limited to specific regions of the brain. Therefore the function of the cerebral cortex over wide brain regions is hardly reflected in DOA measurement. Here, DOA is measured by the quantification of dominant information flow obtained from principle bipartition. Three bipartitioning methods are used to detect the dominant information flow in entire EEG channels and the dominant information flow is quantified by calculating information entropy. High correlation between the proposed measures and the plasma concentration of propofol is confirmed from the experimental results of clinical data in 39 subjects. To illustrate the performance of the proposed methods more easily we present the results for multichannel EEG on a two-dimensional (2D) brain map.

## 1. Introduction

Depth of anesthesia (DOA) should be accurately and adequately maintained in order to prevent potential intraoperative side effects such as hypertension, tachycardia, sweating, lacrimation, increased skeletal muscle tone, and spontaneous movement [1]. For example, the intraoperative awareness due to insufficient anesthesia occurs in 0.1%–0.2% of all surgical patients [2, 3] resulting in significant mental sequela and posttraumatic syndrome [4]. In contrast, anesthetic agent overdose can be a cause of hypotension leading to hypoperfusion of heart and brain in sensitive patients. Due to the individual gap in dose response to the anesthetic agent, adjusting the dose of anesthetic agent to ensure maintenance of the appropriate DOA might remain a considerable burden to the anesthesiologists. Therefore, reliable assessment of DOA is essential in clinical settings.

General anesthesia includes hypnosis as well as analgesia [5]. Several DOA measurements use the features of anesthesia mentioned earlier, including the autonomic nervous system-based methods such as degree of muscle relaxation, hemodynamics, perspiration, and lacrimation [6], as well as the heart rate variability- (HRV-) based method reflecting change in brainstem function [7, 8]. However, little correlation between these parameters and the function of the cerebral cortex, which hardly reflects change in DOA [9, 10], might cause intraoperative awareness. Therefore, the function of the cerebral cortex should be considered in indices for DOA monitoring.

A number of studies are ongoing to develop DOA indices based on cerebral electrical activity. For example, compressed spectral array (CSA) [11] focuses on the change in frequency characteristic of the electroencephalogram (EEG) during anesthesia; spectral edge frequency (SEF) [12], frequency band power ratio [13], or spectral entropy (SpE) [14] measure the change in the pattern of the power spectrum; mid-latency auditory evoked potential (MLAEP) [15] examined the response of the electroencephalogram (EEG) to an auditory stimulus or the bispectral index (BIS) using phase coupling between EEG frequency components [16], the latter of which is currently in clinical use for DOA monitoring [17–19]. However, these previous methods mainly use few EEG channels independently and most of multichannel EEG based studies are limited to specific regions of the brain. This being so, the function of the cerebral cortex over wide brain regions is hardly reflected in DOA measurement. Furthermore, consciousness is associated with functional integration and segregation of the brain [20], and anesthetic-induced unconsciousness is reflected in wide and different regions of the brain [21]. Therefore it needs to consider the function of the cerebral cortex over whole brain regions for DOA monitoring.

In order to overcome these limitations, this study suggests the DOA measurement by analysis of EEG information flow activated from the cerebral cortex over the brain. Information flow in the brain has a dynamic characteristic depending on the condition of the subject; therefore, dominant information flow may reflect consciousness level. In this paper, we focus on the change in dominant information flow occurring during the process of loss of consciousness (LOC) and recovery of consciousness (ROC) for the purpose of DOA measurement. In order to screen the dominant information flow among various information flows existing in multichannel EEGs, 3 indices that bipartition the overall channels into information source groups and target groups are suggested and used to extract the dominant information flow. Then from a perspective of information theory, the quantity of information is measured for quantification of the consciousness level. The representative information entropies for quantifying EEG include mutual information (MI) [22], granger causality [23], and transfer entropy (TE) [24]. TE has been used in DOA studies in only a few cases, despite its excellent performance. The aim of this study was to confirm the potentiality of the proposed methods as an indicator of DOA by quantifying the dominant information flow that reflects the functional activities at the overall cortical areas through information entropy.

## 2. Materials and Methods

### 2.1. Subjects

This study was approved by the Institutional Review Board of the Asan Medical Center (Seoul, South Korea), and written informed consent was obtained in all cases. The study included 39 healthy volunteers over 20 years old and excluded subjects with past history potential to become risk factors upon administration of the study drug in advance, such as cardiovascular, respiratory, kidney, endocrine, hematologic, gastrointestinal, central nervous, or psychiatric disease.

### 2.2. EEG Recordings

EEG was recorded at seven monopolar channels in the frontoparietal regions (Fp1, Fp2, F3, F4, P3, P4, and Cz with the reference electrode in A2 of the international 10–20 system) by a QEEG-8 (Laxtha Inc., Daejeon, Korea) with a sampling frequency of 256 Hz. EEG was continuously recorded from 5 minutes before administration of propofol until 60 minutes after the end of propofol infusion. A ninth-order Butterworth filter was used to remove components above 50 Hz from the EEG signals.

### 2.3. Study Design

Microemulsion propofol (Aquafol™, Daewon Pharm. Co. Ltd., Seoul, Korea) was used as general anesthetics [25]. When the volunteers arrived at the operating theatre, electrocardiography, pulse oximetry, end-tidal carbon dioxide partial pressure, and noninvasive blood pressure monitoring were started and EEG electrodes were applied. An 18 G angiocath was placed in the vein for propofol infusion and a 20 G angiocath was placed in the contralateral radial artery for frequent sampling. Volunteers were preoxygenated with 100% oxygen and then a facial mask through which a supply of 4 L/min of oxygen was applied [26]. The subject groups were classified as 3, 6, and 12 mg/kg/h according to the infusion rate of anesthetic agents, and one dose was designated and injected into each subject for 60 minutes. In order to maximize the safety of the patients, the clinical study was conducted in a consecutive order, starting from the subjects on a low infusion rate (3 mg/kg/h). In order to measure the concentration of propofol, arterial (0.5, 1, 1.5, 2, 3, 4, 6, 8, 10, 15, 20, 30, 40, 50, 58, 60, 62, 66, 70, 80, 90, 120, and 150 min) or venous (180, 240, 300, 600, 720, and 1200 min) blood drawing was conducted, for a total of over 32 times, from before the dose of anesthetics to 20 hours after continuous intravenous infusion. Additionally, the loss of consciousness (LOC) was assessed by giving verbal commands to each subject to open their eyes, immediately after administration of propofol, at an interval of 10 sec until there was no response. In addition, the recovery of consciousness (ROC) was assessed by giving the subjects verbal commands to open their eyes, immediately after the end of the propofol infusion, at an interval of 10 sec until the patient responded [27].

### 2.4. Population Pharmacokinetic Analysis

A population pharmacokinetic analysis was performed with NONMEM VII level 3 (ICON Development Solutions, Ellicott City, MD, USA). Interindividual random variabilities of pharmacokinetic parameters were estimated assuming a log-normal distribution. Diagonal matrices were estimated for the various distributions of *η*, where *η* represented interindividual random variability with a mean of zero and a variance of *ω*^2^. Additive, constant coefficient of variation and combined additive and constant coefficient of variation residual error models were evaluated during the model building process. NONMEM computed the minimum objective function value (OFV), a statistic equivalent to the −2 log likelihood of the model. An *α* level of 0.05, which corresponds to a reduction in the OFV of 3.84 (Chi-square distribution, degree of freedom = 1, *p* < 0.05), was used to distinguish between hierarchical models [28]. One-, two-, and three-compartment disposition models with first-order elimination were tested. The covariates analysed were age, sex (0 = male, 1 = female), weight, height, body surface area [29], body mass index, ideal body weight [30], and lean body mass [31]. Nonparametric bootstrap analysis served to validate the models internally (fit4NM 3.5.1, Eun-Kyung Lee and Gyu-Jeong Noh, http://cran.r-project.org/web/packages/fit4NM/index.html, last access: Oct 17, 2011) [32].

### 2.5. EEG Data Selection

The selection criteria for each EEG index used in this study were as follows: (1) every 30 s during the first 10 min, every 1 min during the second 60 min, after the beginning of the propofol infusion; (2) every 30 s during the first 20 min, every 1 min during the second 20 min, and every 2 min during the third 20 min, after the termination of the propofol infusion [33].

### 2.6. Population Pharmacodynamic Analysis

A sequential modeling approach with post hoc pharmacokinetic estimates was used to derive the population pharmacodynamic parameters. Dissociation between the concentration of propofol and effect of propofol on central nervous system (EEG indices) was linked with an effect compartment. The relationship between the effect-site concentration (*C*_*e*_) of propofol and EEG indices was evaluated using a sigmoid *E*_max_ model as follows:

(1) $$\begin{array}{c} E=E_{0}+\left(\;E_{max}-E_{0}\;\right)\frac{{C_{e}}^{\gamma }}{{C_{e_{50}}}^{\gamma }+{C_{e}}^{\gamma }}, \end{array}$$

where *E* is the each EEG index value, *E*_0_ is the baseline EEG index value when no drug was present, *E*_max_ is the maximum possible drug effect on the EEG index, *C*_*e*_ is the calculated effect-site concentration of propofol, *C*_*e*_50__ is the effect-site concentration associated with 50% of the maximal drug effect on EEG index, and *γ* is the steepness of the effect-site concentration versus EEG index relationship.

### 2.7. Statistics

Prediction probability (*P*_*K*_) was assessed as described by Smith and colleagues [34]. We calculated *P*_*K*_ values using Somers' *D* cross-tabulation statistic on SPSS, which was then transformed from the −1 to 1 scale of Somers' *D* to the 0 to 1 scale of *P*_*K*_ as *P*_*K*_ = 1 − (1 − |Somers'  *D*|) × 2^−1^. The EEG indices and *C*_*e*_ were set as the dependent and independent variables, respectively. Prediction probabilities were calculated using the full measurement set. The standard error (SE) of each *P*_*K*_ was calculated as (SE of Somers' *D*) ×  2^−1^.

## 3. Proposed Methods Quantifying the Depth of Anesthesia

The information flow of a multichannel EEG signal was analyzed for DOA quantification. The information flow was quantified by the representative measuring methods, mutual information [22], and transfer entropy [24], while the efficacy of the DOA measurement was verified by comparison.

### 3.1. Mutual Information

Mutual information measuring the information, shared by both the *X* and *Y* signals, is generally defined as follows:

(2) $$\begin{array}{c} MI\left(\;X;Y\;\right)=\underset{x,y}{\sum }p\left(\;x,y\;\right)\ln\frac{p\left(x,y\right)}{p\left(x\right)p\left(y\right)}, \end{array}$$

where *p*(*x*, *y*) is the joint probability distribution function of *X* and *Y* and *p*(*x*) and *p*(*y*) are the marginal probability distribution function of *X* and *Y*, respectively. This equation can be expressed as sum of Shannon entropies.

(3) $$\begin{array}{c} MI\left(\;X;Y\;\right)=H\left(\;X\;\right)+H\left(\;Y\;\right)-H\left(\;X,Y\;\right). \end{array}$$

When defining the EEG signals obtained from two different regions of the brain as *X* and *Y*, (3) can be used to quantify the degree of information shared by the two regions. The amount of information shared by various regions can be confirmed by applying MI to multichannel EEG signals and we can observe the information flow in the overall cortical areas.

### 3.2. Transfer Entropy

Transfer entropy is the measurement used to quantify the effect of information obtained from the *Y* (*y*_*n*_) signal at a specific time *n* on the *X* (*x*_*n*+1_) signal at a future time. When defining each probability distribution for *x*_*n*_ and *y*_*n*_ as *p*(*x*_*n*_) and *p*(*y*_*n*_), respectively, TE from *Y* to *X*, *T*_*Y*→*X*_, is defined as follows:

(4) $$\begin{array}{c} T_{Y\to X}=\underset{x,y}{\sum }p\left(\;x_{n+1},x_{n}^{\left(k\right)},y_{n}^{\left(k\right)}\;\right)\ln\frac{p\left(x_{n+1}\;|\;x_{n}^{\left(k\right)},y_{n}^{\left(k\right)}\right)}{p\left(x_{n+1}\;|\;x_{n}^{\left(k\right)}\right)}. \end{array}$$

*x*_*n*_^(*k*)^ denotes [*x*_*n*−(*k*−1)_, *x*_*n*−2(*k*−1)_,…, *x*_*n*−*d*(*k*−1)_] where *d* is the embedding dimension and *k* is the embedding delay. We applied the uniform embedding scheme [35] to evaluate TE and we fixed the embedding dimension and the embedding delay at 1 for computational reasons [24]. TE in (4) can be obtained as follows:

(5) TB→AHAn+1 ∣ An−HAn+1 ∣ An,Bn=HAn+1,An−HAn−HAn+1,An,Bn−HAn,Bn,

where *A* is a target group and *B* is a source group. For a multivariate random vector, *A*, assuming the Gaussian p.d.f. in [36], the Shannon entropy can be calculated as

(6) $$\begin{array}{c} H\left(\;A\;\right)=\frac{1}{2}\ln{\left(\;2\pi e\;\right)}^{k}\left|\;\Sigma_{A}\;\right|, \end{array}$$

where *k* is a number of random variables, Σ_*A*_ is covariance matrix of *A*, and |·| denotes the matrix determinant. For example, if *A* consists of two channels, *X* and *Y*, and *B* consists of a channel *Z*, then *H*(*A*_*n*+1_, *A*_*n*_, *B*_*n*_) is expressed as

(7) $$\begin{array}{c} H\left(\;A_{n+1},A_{n},B_{n}\;\right)=\frac{1}{2}\ln{\left(\;2\pi e\;\right)}^{k}\left|\;\Sigma_{A_{n+1},A_{n},B_{n}}\;\right|, \end{array}$$

where Σ_*A*_*n*+1_,*A*_*n*_,*B*_*n*__ = cov(*V*_*A*_*n*+1_,*A*_*n*_,*B*_*n*__), $V_{A_{n+1},A_{n},B_{n}}=\left[\begin{array}{ccc} A_{n+1} & A_{n} & B_{n} \end{array}\right]$ and *A*_*n*+1_, *A*_*n*_, and *B*_*n*_ are as follows:

(8) An+1=x2y2x3y3⋮⋮xNyN,An=x1y1x2y2⋮⋮xN−1yN−1,Bn=z1z2⋮zN−1.

Similarly, *H*(*A*_*n*+1_, *A*_*n*_), *H*(*A*_*n*_), and *H*(*A*_*n*_, *B*_*n*_) can be obtained.

When defining the EEG signal obtained from the *B* region at a specific time point *n* as *y*_*n*_ and defining the EEG signal obtained from the *A* region at the future time point *n* + 1 as *x*_*n*+1_, (5) can be used for quantifying the effect of information from the *B* region on the creation of future information from the *A* region. In contrast, *T*_*A*→*B*_ represents the effect of information from the *A* region on the creation of future information from the *B* region. Accordingly, although MI represents the amount of shared information that is not directional in the information flow between two regions, TE refers to the degree of information transfer considering the directional nature of information flow between two regions. In [35], the simple value of TE has been used to exploit the mechanisms in EEG between patients with disorders of consciousness.

### 3.3. Mutual Information versus Transfer Entropy

**Figure 1:**
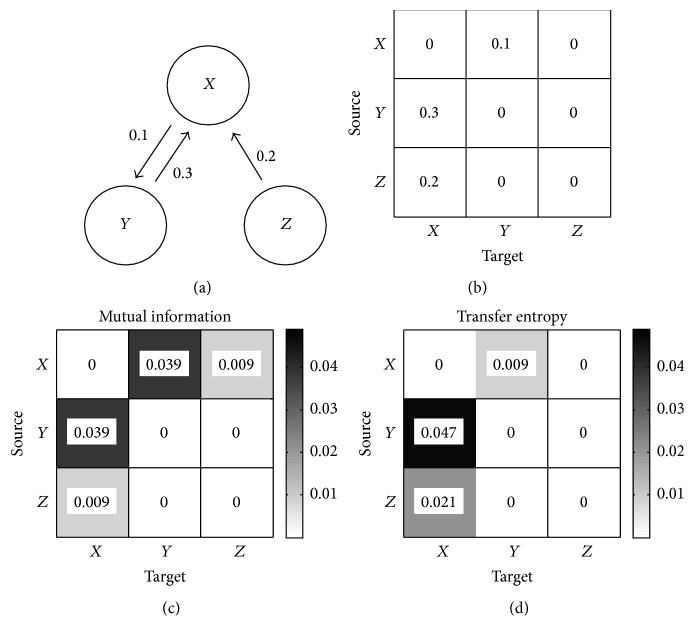
Comparison of mutual information and transfer entropy. (a) System model, (b) connection strength, *F*_*i*,*j*_, (c) mutual information of system, and (d) transfer entropy of system.

An arbitrary system, including information flow as shown in Figure 1(a), was set for a characteristic comparison between MI and TE. Arrows indicate the strength of information flows linking *X* to *Y* or *Z* and vice versa. The channel activities *x*_*t*_, *y*_*t*_, and *z*_*t*_ are generated by vector autoregression (VAR) [36] as follows:

(9) xt=F1,1xt−1+F1,2yt−1+F1,3zt−1+e1,t,yt=F2,1xt−1+F2,2yt−1+F2,3zt−1+e2,t,zt=F3,1xt−1+F3,2yt−1+F3,3zt−1+e3,t,

where *F*_*i*,*j*_ is the connection strength from the source *i* to the target *j* as in Figure 1(b) and *e*_*i*,*t*_ is the independent Gaussian noise. The current (time *t*) observation of each channel depends on its own lagged values as well as on the lagged values of other channels.

Figures 1(c) and 1(d) show the results of the quantification of information flow by MI and TE, respectively. MI shows the symmetric result with no division of the source and the target in Figure 1(c), while TE reflects the directional information flow between channels as confirmed in Figure 1(d). For example, the *X* → *Z* with no information flow has a TE value of 0, while MI has 0.009 of the shared information because MI neither contains dynamical nor directional information [24]. Furthermore, it is confirmed that TE more effectively represents the amount of information occurring between channels than MI. In other words, TE is more useful than MI in the quantification of information flow with consideration of directionality.

**Figure 2:**
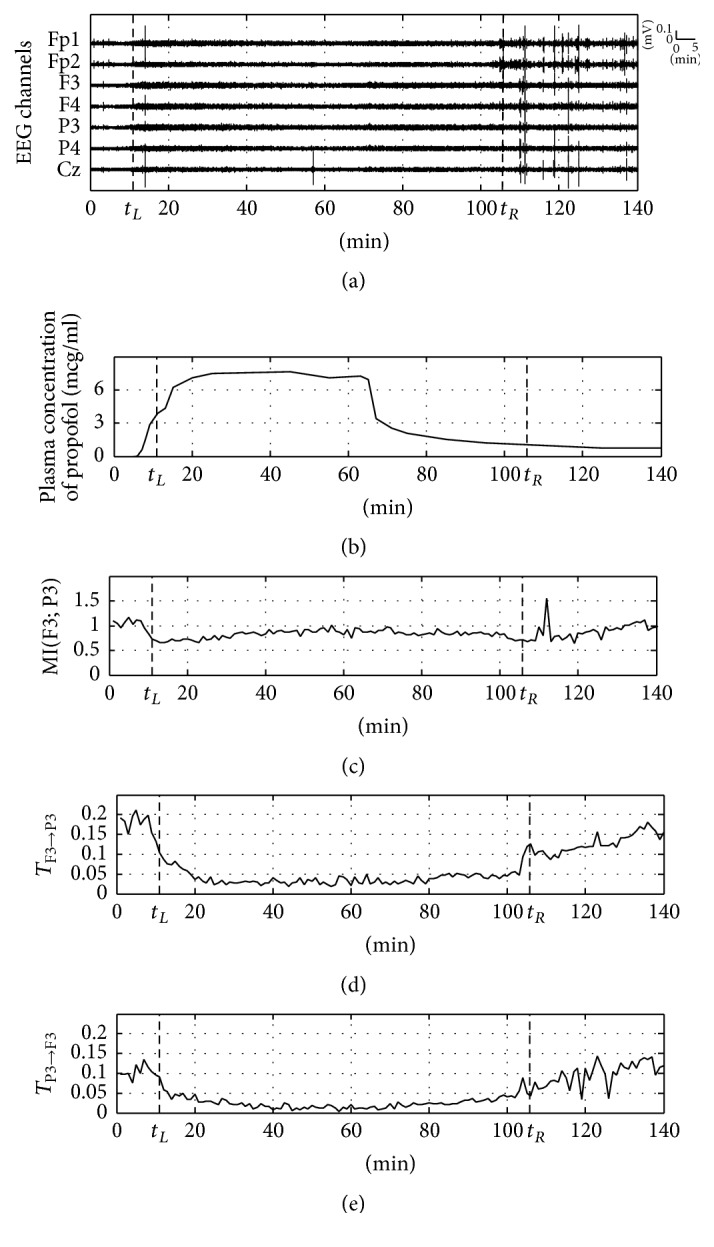
Example of mutual information and transfer entropy using clinical EEG data (the two vertical dashed lines indicate *t*_*L*_ and *t*_*R*_ corresponding to LOC and ROC, resp.). (a) Raw multichannel EEGs for 140 min before and after anesthesia, (b) plasma concentration of propofol (infusion rate = 12 mg/kg/h), (c) mutual information between frontal lobe (F3) and parietal lobe (P3), (d) transfer entropy in feedback pathway (F3 → P3), and (e) transfer entropy in feedforward pathway (P3 → F3).

Figure 2 shows the result of MI and TE for the clinical EEG data. A change in the information flow of the EEG signal was observed for two channels, F3 and P3, which are applied to the frontal lobe and parietal lobe, respectively, among a total of 7 EEG channels (Fp1, Fp2, F3, F4, P3, P4, and Cz) used in the experiment. TE value was calculated by using the EEG data for 60 seconds at a 30-second interval, and the time delay of TE is 7.8 ms.  Figure 2(a) shows the multichannel EEG for a total of 140 minutes before and after anesthesia; two vertical dashed lines, *t*_*L*_ and *t*_*R*_, indicate the time points of loss of consciousness (LOC) and recovery of consciousness (ROC), respectively.  Figure 2(b) shows the plasma concentration of propofol over time, which confirms the time point of anesthetic agent infusion and change in anesthetic concentration. It is difficult to observe the change in information flow around the LOC and ROC in Figure 2(a), only with the raw EEG signal, and DOA cannot be confirmed.  Figure 2(c) shows the result of MI for the EEG signal of the frontal lobe (F3) and parietal lobe (P3). The figure shows a slight decrease in the EEG signal immediately after the infusion of the anesthetic agent; however, it does not reflect the change in anesthetic concentration well, as there is little change in the figure thereafter. Figures 2(d) and 2(e) show the result of TE for the information flow of the feedback pathway from the frontal lobe to the parietal lobe (F3 → P3) and the feedforward pathway from the parietal lobe to the frontal lobe (P3 → F3), respectively.  Figure 2(d) shows the remarkable trend of the dramatically decreased figure of *T*_F3→P3_ at the anesthetic infusion and the reincreased figure after ROC, clearly reflecting the change in anesthetic concentration. This result is consistent with the result of previous studies [27, 37, 38], in which loss of consciousness is accompanied by a reduction in frontoparietal feedback connectivity. *T*_P3→F3_ in Figure 2(e) presents a small figure and low decrease rate compared to *T*_F3→P3_, but the pattern of the overall change per anesthetic concentration is similar to *T*_F3→P3_. In this example, TE considering directionality of information flow is more appropriate for EEG analysis for DOA measurement than MI.

### 3.4. Proposed Methods

Although anesthesia relies on the interconnection of various cortical areas, most previous DOA studies used few EEG channels independently or targeted a specific region of the brain. Thus, an accurate DOA is hardly expected by EEG analysis for the specific cortical area.

**Figure 3:**
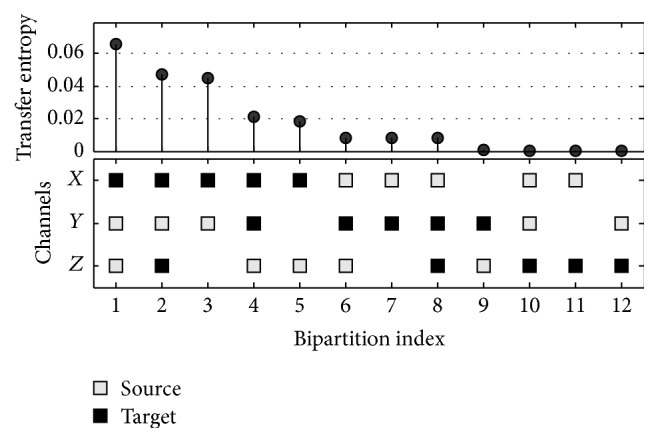
Transfer entropy according to various bipartitions of the system shown in Figure 1.

Figure 3 shows the result of TE according to various channel combinations for the system in Figure 1. When channels are bipartitioned into the source and the target, 12 combinations exist. In Figure 3, axis *x* indicates the index of 12 bipartitions, while axis *y* is TE value of the applicable combination. This suggests there could be a considerable difference in TE depending on the selection of the source and the target.

Information flow is observed for the multichannel EEG signal according to various combinations between channels, while the EEG analysis of specific channel combinations only provides the characteristic of the applicable regions. In order to compensate for this limitation, we divide EEG channels into two subgroups where the considerable information flow occurs between them. We call this partition as the* principle bipartition*. Then the information in terms of TE in the principle bipartition is used for a measure reflecting the major characteristics of various cortical areas. In other words, DOA is measured by the quantification of the dominant information flow, which is calculated in terms of TE in the principle bipartition. For the principle bipartition, three criteria based on TE are used: the maximum (*T*_max_), the minimum (*T*_min_), and the mean (*T*_mean_) of TE. The maximum information flow with the largest value of TE might be the first consideration for the selection of the principle bipartition. This is applicable to the first bipartition index in Figure 3, of which *Y* and *Z* are the source and *X* is the target. As confirmed in the system in Figure 1(a), the largest amount of information flow occurs in the case where *Y* and *Z* are the source and *X* is the target. Also, the minimum information bipartition (MIB) [39, 40] recently published can be used as another principle bipartitioning. The MIB divides channels into two subgroups so that they have the minimum information flow. Then among these partitions, the partition which has the largest information is selected. Although this method cannot avoid the directed or mediated influences between channels, it is suitable for reflecting the functional integration and segregation of the brain which is associated with consciousness. Finally, the mean TE value of all possible bipartitions was used as the third index, *T*_mean_.

**Figure 4:**
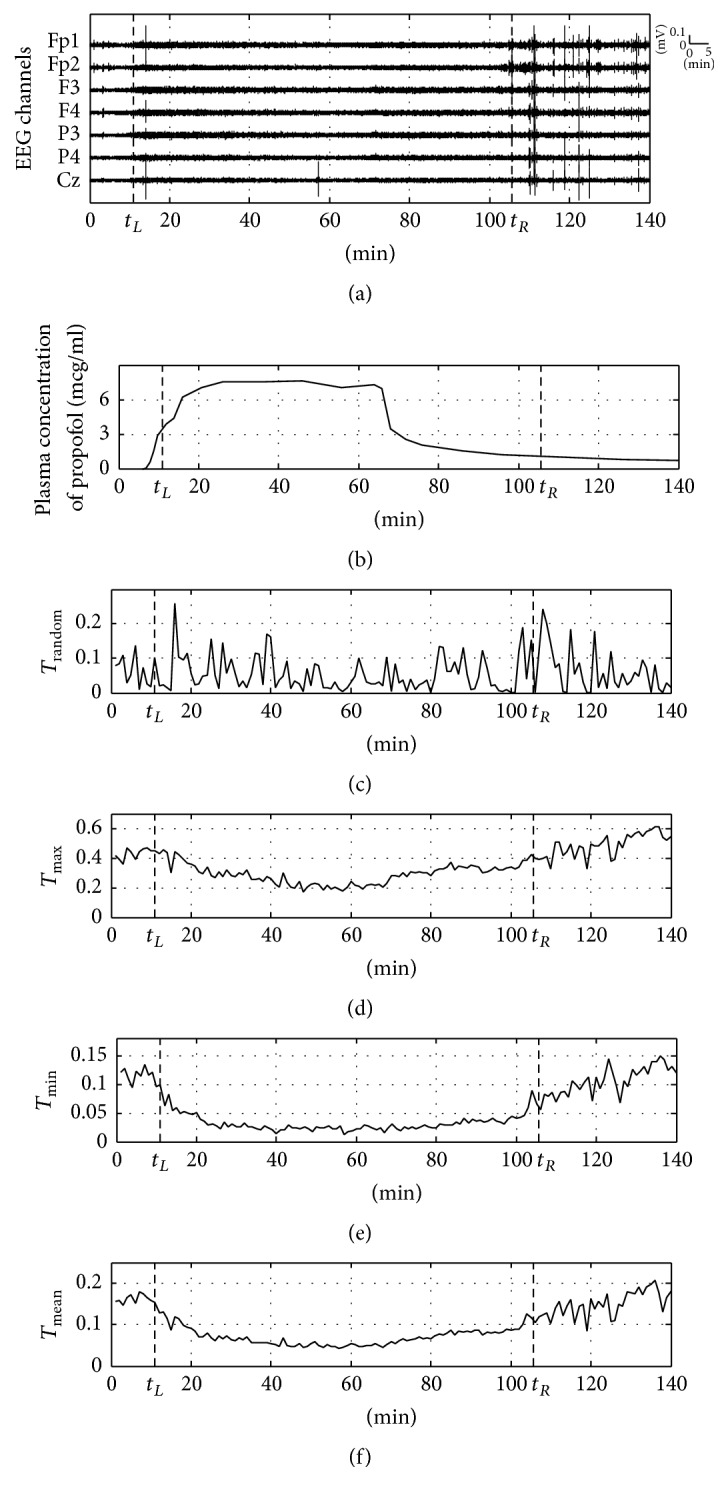
Transfer entropy of various bipartitions in clinical multichannel EEGs. (a) Raw multichannel EEGs for 140 min before and after anesthesia, (b) plasma concentration of propofol (infusion rate = 12 mg/kg/h), (c) transfer entropy for arbitrary bipartition, (d) transfer entropy for principle bipartition with maximum information flow, (e) transfer entropy for principle bipartition with MIB, and (f) averaged transfer entropy for all possible bipartitions.

*T*_max_, *T*_min_, and *T*_mean_ examined how well these reflect the activities of the overall cortical areas.  Figure 4 shows TE results of the actual EEG data for various bipartitioning methods. Figures 4(a) and 4(b) show the same figure for the EEG signal as that in Figure 2(a) and the plasma concentration of propofol in Figure 2(b), respectively.  Figure 4(c) shows TE result of arbitrary bipartitioning. It does not reflect the change in anesthetic concentration with large variation.  Figure 4(d) shows TE result of the principle bipartition, which has the maximum information flow, *T*_max_. The trend is observed whereby TE decreases after LOC and reincreases at the discontinuation of the anesthetics infusion, after approximately 65 minutes, and is inversely proportional to the change in plasma concentration of propofol.  Figure 4(e) shows TE result of the principle bipartition by MIB, *T*_min_. A definite trend is observed whereby TE dramatically decreases immediately after anesthetics infusion and reincreases around ROC. When compared to Figure 4(d), the overall values are low, but the change in anesthetic concentration is clearly reflected.  Figure 4(f) shows the mean TE results of all bipartitions, *T*_mean_. These results are very similar to the pattern of the changes in Figures 4(d) and 4(e), and *T*_mean_ which also clearly reflects the change in anesthetic concentration. Through comparison between the results of the proposed methods and Figure 4(c), it is confirmed that the way in which bipartitioning is performed is one of the important factors in the quantification of DOA. In addition, *T*_max_, *T*_min_, and *T*_mean_ as indices reflecting information flow of all the cortical areas can be used for measuring DOA by reflecting brain activities from various regions.

## 4. Experimental Results

The efficacy of the proposed methods was confirmed in a total of 39 subjects. Overall, the subjects are classified into 3 groups according to the infusion rate of the anesthetic agent (3, 6, and 12 mg/kg/h) and each group consists of 13 subjects.

**Figure 5:**
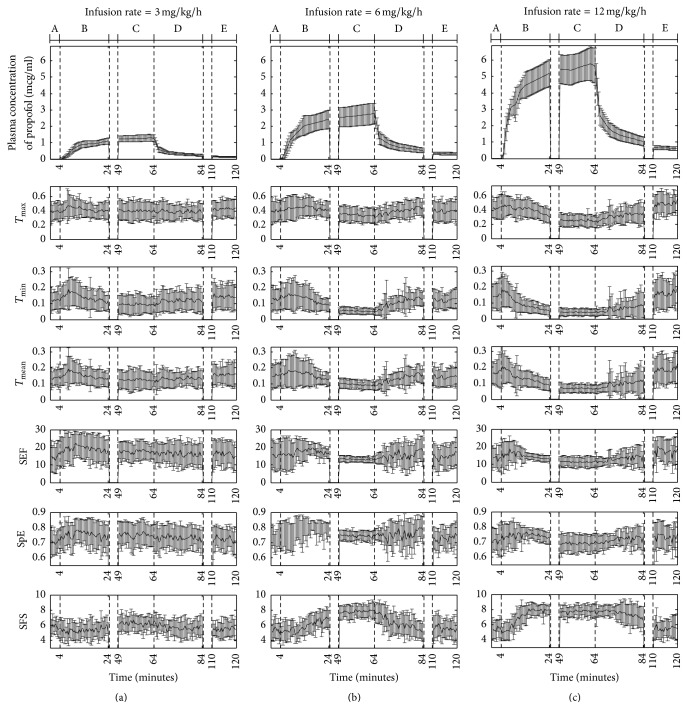
Results of proposed indices and conventional indices averaged over 13 subjects for three infusion rates (error bar denotes the standard deviation over 13 subjects. A: before anesthetics infusion, B: increase in anesthetic concentration, C: steady state in anesthetic concentration, D: decrease in anesthetic concentration, and E: near ROC and recovery). (a) Results for infusion rate of 3 mg/kg/h, (b) results for infusion rate of 6 mg/kg/h, and (c) results for infusion rate of 12 mg/kg/h.

Figure 5 shows the results of the proposed and conventional indices such as spectral edge frequency (SEF) [12], spectral entropy (SpE) [14], and synch fast slow (SFS) [16] according to the infusion rate in the subjects. The EEG signal which derived from one channel of prefrontal lobe (Fp1) was used for calculating conventional indices and 7 EEG channels (Fp1, Fp2, F3, F4, P3, P4, and Cz) were used for proposed indices. The recoding intervals were divided into 5 sections (A–E) according to the change in plasma concentration of propofol: section A is the preanesthetics infusion phase (4 minutes); section B is the increasing phase in which the anesthetic concentration dramatically increases before and after postanesthetics infusion LOC (20 minutes); section C is the maintenance phase in which a high level of anesthetic concentration is relatively and consistently maintained (15 minutes); section D is the decreasing phase in which the anesthetic concentration dramatically decreases after discontinuation of anesthetics infusion (20 minutes); and section E is the recovery phase in which the anesthetic concentration is eventually recovered after ROC (10 minutes). The error bar denotes the standard deviation over 13 subjects.  Figure 5(a) shows the result of the EEG indices for the subjects in which an infusion rate of 3 mg/kg/h was applied. In this case, TE value is relatively consistent, regardless of the change in anesthetic concentration and no noticeable change is observed. And the conventional indices also do not show significant changes. Meanwhile, a noticeable trend is observed of a change in TE values depending on the change in plasma concentration of propofol shown in Figure 5(b) with an infusion rate of 6 mg/kg/h. First, a tendency is observed of decreased TE values after LOC in section B, whereby anesthetic concentration increases, while TE values are maintained as the lowest level among all the sections, while a smaller standard deviation than the other sections is observed in section C. In addition, TE values increase in inverse proportion to the plasma concentration of propofol in section D, in which the anesthetic concentration dramatically decreases due to the discontinuation of anesthetics infusion; these values are then recovered to the level of the preanesthesia phase in section E. The range of change in TE values for *T*_max_ shown in the second box is relatively smaller than the range of change in the other two proposed indices, but the general graphic change patterns are similar. On the other hand, the conventional indices, SEF_95_ and SpE_0.8–47 Hz_, do not change proportionally to the plasma concentration of propofol. But the SFS presented in Figure 5(b) increases in proportion to the anesthetic concentration.  Figure 5(c) shows the result of the EEG indices for the subjects in which an infusion rate of 12 mg/kg/h was used. The deep sedation is determined from the plasma concentration of propofol shown in the box at the top of the figure. In this case, the decreasing rate of TE proportional to the increase of anesthetic concentration in the proposed methods is significant and the decreasing gradient is also considerable in section B. In addition, the radical decrease of TE immediately after anesthetics infusion differs from that shown in Figure 5(b).

This result shows that as the infusion rate is increased the decreasing rate of TE also increases. Such change is found to occur immediately after anesthetics infusion, while the mean TE in section C is much lower. Meanwhile, the increasing rate of TE in section D is even slower at an infusion rate of 12 mg/kg/h than at 6 mg/kg/h, which means that as the infusion rate is increased, the rate of consciousness recovery decreases. Therefore, the proposed methods effectively reflect not only the quantification of anesthetic concentration over time but also the change in consciousness level per infusion rate.

**Figure 6:**
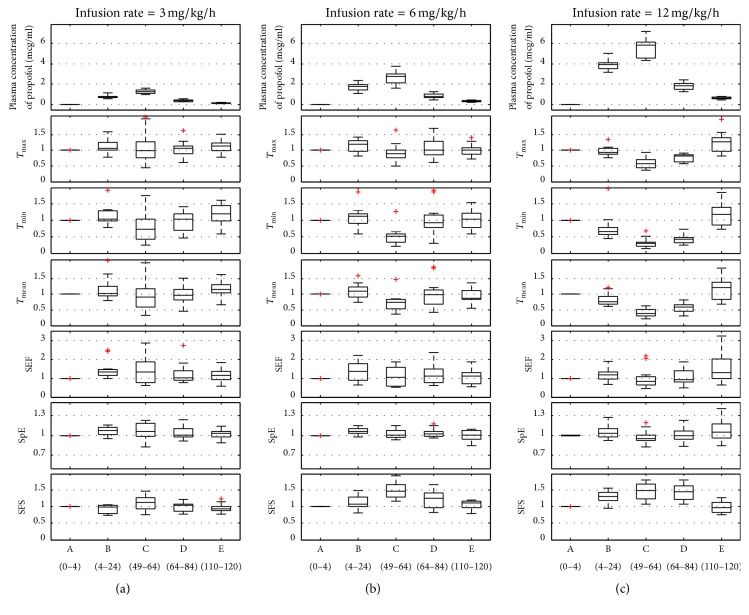
Boxplots of the normalized results of the EEG indices. The middle line is the median value of 13 subjects. (a) Normalized value of indices for infusion rate of 3 mg/kg/h, (b) normalized value of indices for infusion rate of 6 mg/kg/h, and (c) normalized value of indices for infusion rate of 12 mg/kg/h.

In order to quantitatively compare various indices and remove subject dependency, the results of the indices were normalized with the mean value of section A for each subject, which are shown in Figure 6. The boxplots of all indices at five sections are expressed with median values and quartiles (25%–75%) of averaged values for each section, respectively.

### 4.1. Pearson's Correlation Coefficient

The Pearson's correlation coefficients between the EEG indices and the plasma concentration of propofol are shown in Table 1. A total of 130 EEG data points were used to determine the correlation coefficients of each subject during anesthesia (B-C) and recovery (D-E). Each correlation value in Table 1 shows the mean and the standard deviation of 13 subjects and the *p* value is also averaged over 13 subjects. Bold indicates the highest and the second highest correlations in each infusion rate. The values of the correlation coefficients for all the EEG indices are not high at the infusion rate of 3 mg/kg/h, but these values of the proposed indices are higher than conventional indices at the rate of 12 mg/kg/h. Particularly, in the case of *T*_min_, the mean value of correlation coefficients is significantly high (−0.815, *p* < 0.001).

### 4.2. Population Pharmacokinetic Analysis

In total, 1,017 plasma concentration measurements from 36 healthy volunteers (male : female = 1 : 1) were used to characterize the pharmacokinetics of propofol. A three-compartment mammillary model best described the pharmacokinetics of propofol.  Table 2 presents the notion that the population pharmacokinetic parameter estimates and the results of nonparametric bootstrap replicates of the final pharmacokinetic model of propofol.

### 4.3. Population Pharmacodynamic Analysis

A total of 5,076 EEG data points were used to determine the pharmacodynamic characteristics of each EEG index. A sigmoid *E*_max_ model well described the time course of observed EEG indices values. Population pharmacodynamic parameter estimates and interindividual variability of the pharmacodynamic models are shown in Table 3.

### 4.4. Prediction Probability and Spearman's Correlation Coefficient

*P*_*K*_ values and Spearman's correlation coefficients of the EEG indices are shown in Table 4. *P*_*K*_ values were largest and Spearman's correlation coefficients were second-largest in *T*_min_, which indicates that *T*_min_ is appropriate for the assessment of the propofol effect on the electroencephalogram.

### 4.5. Two-Dimensional Brain Map

**Figure 7:**
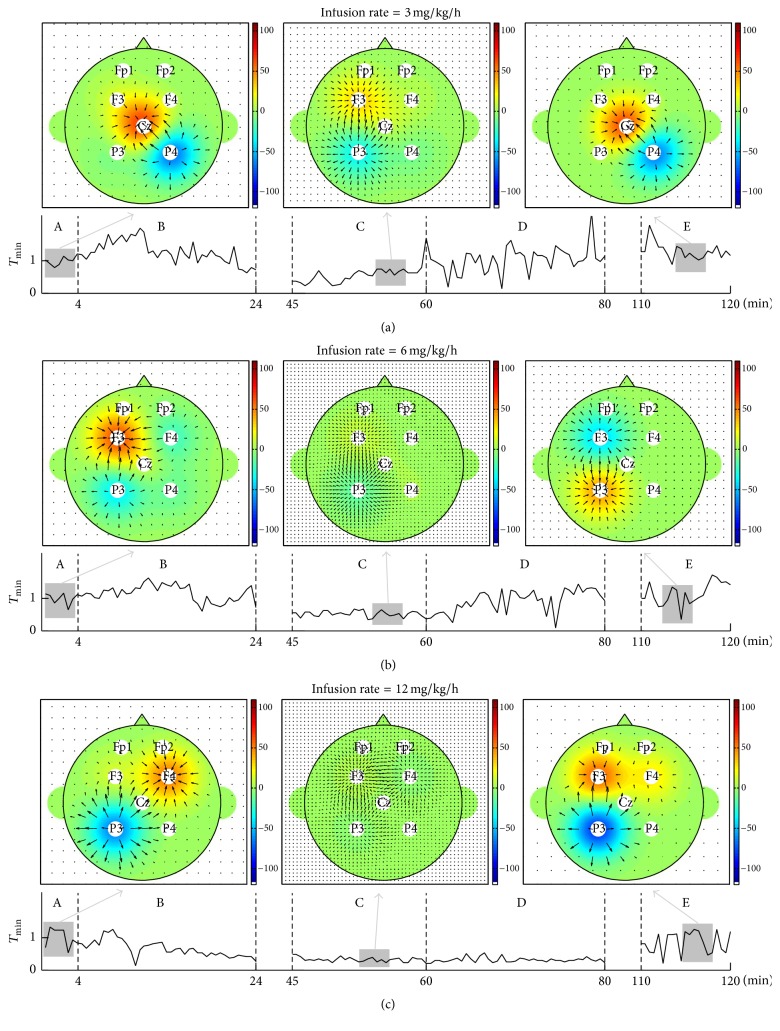
2D visualization of dominant information flow using the proposed method, *T*_min_. The size and the direction of arrows are proportional to the information flow (blue: information source, red: information target). (a) *T*_min_ for infusion rate of 3 mg/kg/h, (b) *T*_min_ for infusion rate of 6 mg/kg/h, and (c) *T*_min_ for infusion rate of 12 mg/kg/h.

The other advantage of the proposed methods is the directional information flow in multichannel EEGs. To illustrate the performance of the proposed indices more easily we have presented the results for multichannel EEG on a two-dimensional (2D) brain map.  Figure 7 shows a 2D visualization of the dominant information flow using *T*_min_. The dominant information flows in sections A, C, and E were compared. The dominant information flow at each section is expressed as the mean *T*_min_ for 4 minutes. The size of the arrow is proportional to the value, while the direction of the arrow refers to the direction of information flow. The blue area indicates the source channel group, while the red area indicates the target channel group. Figures 7(a)–7(c) show the change in dominant information flow according to the three infusion rates, where the dominant information flow according to the anesthetic concentration is easily detected and the DOA is more intuitionally understood.

## 5. Conclusions

The change in consciousness level before and after anesthesia was examined by quantification of information flow of a multichannel EEG. Three bipartitioning methods used to detect the dominant information flow in entire channels were suggested. *T*_max_, *T*_min_, and *T*_mean_ were suggested as the indices for the three bipartitions. The proposed methods as indices reflecting the activities of cerebral cortex in overall cortical areas are distinctive from other previous analysis methods limited to the specific region. High correlation between the proposed measures and the plasma concentration of propofol was confirmed from the experimental results of clinical data in 39 subjects; that is, as the infusion rate was increased, the change in consciousness level before and after anesthesia increased. In particular, in the case of deep sedation, it is confirmed that the loss of consciousness progresses rapidly by anesthetics infusion and the recovery rate is slow. From these results, the potentiality of the proposed methods for the DOA indices can also be confirmed. Furthermore, we have evaluated the results in terms of the prediction probability (*P*_*K*_) and Spearman's correlation coefficients between the effect-site concentration of propofol and the various EEG indices. Here a population pharmacokinetic analysis was performed with NONMEM VII level 3 and nonparametric bootstrap analysis served to validate the model. Recently a new combined theoretical model based on a pharmacokinetics and a neural mass model (NMM) was presented aiming at simulating EEG during propofol-induced anesthesia [41]. It would better to validate the results with the new model, PK-NMM. The limitation of the validation remains for future study with more rigorous analysis. In addition, these results are presented on the 2D brain map using the characteristics of principle bipartitioning comprising the target and the source and the change in DOA was easily and intuitionally understood.

## Figures and Tables

**Table 1:** Pearson's correlation coefficients between the estimation of anesthesia depth by EEG indices and the plasma concentration of propofol during anesthesia and recovery phase (mean ± std (*p* value)).

Phase	Index	Infusion rate		
		3	6	12
Anesthesia (B-C)	*T*_max_	−0.150 ± 0.547 (*p* = 0.018)	−0.250 ± 0.410 (*p* = 0.080)	−0.615 ± 0.180 (*p* = 0.004)
	*T*_min_	−0.382 ± 0.480 (*p* = 0.034)	−0.566 ± 0.344 (*p* = 0.081)	−0.815 ± 0.126 (*p* < 0.001)
	*T*_mean_	−0.298 ± 0.546 (*p* = 0.047)	−0.478 ± 0.407 (*p* = 0.114)	−0.764 ± 0.130 (*p* < 0.001)
	SEF	−0.109 ± 0.492 (*p* = 0.158)	−0.116 ± 0.584 (*p* = 0.053)	−0.366 ± 0.334 (*p* = 0.073)
	SpE	−0.029 ± 0.390 (*p* = 0.262)	0.020 ± 0.426 (*p* = 0.161)	−0.237 ± 0.387 (*p* = 0.170)
	SFS	0.264 ± 0.406 (*p* = 0.040)	0.635 ± 0.141 (*p* = 0.003)	0.646 ± 0.147 (*p* = 0.015)

Recovery (D-E)	*T*_max_	−0.134 ± 0.381 (*p* = 0.137)	−0.253 ± 0.314 (*p* = 0.267)	−0.520 ± 0.147 (*p* = 0.020)
	*T*_min_	−0.292 ± 0.342 (*p* = 0.097)	−0.434 ± 0.372 (*p* = 0.120)	−0.558 ± 0.121 (*p* = 0.015)
	*T*_mean_	−0.241 ± 0.352 (*p* = 0.081)	−0.372 ± 0.346 (*p* = 0.168)	−0.556 ± 0.147 (*p* = 0.027)
	SEF	0.035 ± 0.262 (*p* = 0.209)	−0.100 ± 0.290 (*p* = 0.249)	−0.362 ± 0.229 (*p* = 0.066)
	SpE	0.041 ± 0.282 (*p* = 0.434)	0.017 ± 0.240 (*p* = 0.337)	−0.241 ± 0.267 (*p* = 0.198)
	SFS	0.116 ± 0.327 (*p* = 0.191)	0.413 ± 0.229 (*p* = 0.131)	0.408 ± 0.165 (*p* = 0.074)

Bold indicates the highest and the second highest correlations in each infusion rate.

**Table 2:** Population pharmacokinetic parameter estimates, interindividual variability, and median parameter values (2.5–97.5%) of the nonparametric bootstrap replicates of the final pharmacokinetic model of propofol.

Parameters		Estimates (RSE, %)	CV (%)	Median (2.5–97.5%)
*V*_1_ (L) = *θ*_1_ × (LBM/48)	*θ*_1_	17.5 (6.6)	32.9	17.4 (15.3–19.8)
*V*_2_ (L)		96.3 (5.9)	—	96.5 (84.4–108)
*V*_3_ (L)		1460 (3.2)	—	1430 (1015–1500)
Cl (L/min)		1.13 (3.7)	19.4	1.15 (1.06–1.28)
*Q*_1_ (L/min)		1.03 (4.6)	—	1.035 (0.944–1.13)
*Q*_2_ (L/min)		0.894 (4.2)	18.1	0.878 (0.789–0.955)
*σ*		0.0912 (6.5)	—	0.091 (0.079–0.102)

A log-normal distribution of interindividual random variability was assumed. Residual random variability was modeled using constant CV error model. Nonparametric bootstrap analysis was repeated 1,000 times. RSE: relative standard error = SE/mean × 100 (%). LBM: lean body mass calculated using the Janmahasatian formula [31]. *V*_1_: central volume of distribution (Vd), *V*_2_: rapid peripheral Vd, *V*_3_: slow peripheral Vd, Cl: metabolic clearance, *Q*_1_: intercompartmental clearance between central and rapid peripheral compartments, and *Q*_2_: intercompartmental clearance between central and slow peripheral compartments.

**Table 3:** Population pharmacokinetic parameter estimates and interindividual variability of the pharmacokinetic models of propofol.

Indices	*E*_0_	*E*_max_	*C*_*e*_50__	*γ*	*k*_*e*0_
*T*_max_	0.248	0.177	1.15	3.93	0.0145
	(16.4, 70.1)	(12.3, 44.9)	(19.7, 78.9)	(8.7, 136.4)	(0.8, 199.8)
*T*_min_	0.138	0.0387	1.04	5.86	0.155
	(10.9, 52.7)	(6.7, 56.8)	(10.8, 47.0)	(27.3, 113.1)	0.155 (17.9, 75.3)
*T*_mean_	0.12	0.0753	1.0	2.44	0.138
	(0.1, 60.6)	(0.5, 48.0)	(0.3, 56.7)	(1.7, 126.1)	(1.1, 87.4)
SEF	16.9	10.1	1.0	3.07	0.09
	(15.3, 51.3)	(15.0, 52.5)	(9.8, 56.1)	(14.5, 78.7)	(12.4, 77.5)
SpE	0.745	0.632	1.04	5.04	0.101
	(1.8, 8.4)	(1.6, 21.5)	(3.8, 65.4)	(31.2, 164.3)	(0.8, 137.5)
SFS	5.23	6.19	0.967	4.69	0.201
	(3.5, 14.6)	(2.7, 26.4)	(10.0, 41.4)	(0.1, 163.7)	(27.8, 92.4)

Data are expressed estimate (RSE, % CV). A log-normal distribution of interindividual random variability was assumed. Residual random variability was modeled using additive error model. RSE: relative standard error = SE/mean × 100 (%). SE: standard error.

**Table 4:** Prediction probability (*P*_*K*_) values and Spearman's correlation coefficients between *C*_*e*_ of propofol and the EEG indices.

Indices	*P*_*K*_ (SE, 95% CI)	Spearman's corr. coeff.
*T*_max_	0.6207 (0.0046, 0.6117–0.6297)	−0.349 (*p*<0.001)
*T*_min_	0.7191 (0.0037, 0.7119–0.7263)	−0.607 (*p*<0.001)
*T*_mean_	0.6891 (0.0041, 0.6811–0.6972)	−0.527 (*p*<0.001)
SEF	0.6034 (0.0046, 0.5946–0.6128)	−0.289 (*p*<0.001)
SpE	0.5671 (0.0046, 0.5582–0.5760)	−0.189 (*p*<0.001)
SFS	0.2830 (0.0032, 0.2767–0.2892)	0.643 (*p*<0.001)

SE: standard error and CI: confidence interval.
